# Roles of Autophagy in Oxidative Stress

**DOI:** 10.3390/ijms21093289

**Published:** 2020-05-06

**Authors:** Hyeong Rok Yun, Yong Hwa Jo, Jieun Kim, Yoonhwa Shin, Sung Soo Kim, Tae Gyu Choi

**Affiliations:** 1 Department of Biomedical Science, Graduate School, Kyung Hee University, Seoul 02447, Korea; foryou018@khu.ac.kr (H.R.Y.); jac03032@khu.ac.kr (Y.S.); 2 Biomedical Science Institute, Kyung Hee University, Seoul 02447, Korea; yonghwa.jo@khu.ac.kr (Y.H.J.); popje124@khu.ac.kr (J.K.); 3 Department of Biochemistry and Molecular Biology, School of Medicine, Kyung Hee University, Seoul 02447, Korea

**Keywords:** autophagy, reactive oxygen species, oxidative stress

## Abstract

Autophagy is a catabolic process for unnecessary or dysfunctional cytoplasmic contents by lysosomal degradation pathways. Autophagy is implicated in various biological processes such as programmed cell death, stress responses, elimination of damaged organelles and development. The role of autophagy as a crucial mediator has been clarified and expanded in the pathological response to redox signalling. Autophagy is a major sensor of the redox signalling. Reactive oxygen species (ROS) are highly reactive molecules that are generated as by-products of cellular metabolism, principally by mitochondria. Mitochondrial ROS (mROS) are beneficial or detrimental to cells depending on their concentration and location. mROS function as redox messengers in intracellular signalling at physiologically low level, whereas excessive production of mROS causes oxidative damage to cellular constituents and thus incurs cell death. Hence, the balance of autophagy-related stress adaptation and cell death is important to comprehend redox signalling-related pathogenesis. In this review, we attempt to provide an overview the basic mechanism and function of autophagy in the context of response to oxidative stress and redox signalling in pathology.

## 1. Introduction

Autophagy (self-eating) was first introduced by Christian de Duve in 1963 as a lysosome-mediated disposal process [1]. Autophagy is a catabolic process that is essential for cellular homeostasis through the removal of cellular molecules, such as protein aggregates and damaged organelles, via lysosomal digestion [2,3]. Principally, it regulates the balance between organelle biogenesis, protein synthesis and the clearance of cells [4], which is involved in cellular remodelling during development and differentiation [5]. Autophagy occurs under conditions of glucose or amino acid deprivation, oxidative stress, hypoxia and exposure to xenobiotics [6]. Autophagy has emerged as a critical mediator of pathological responses is associated with reactive oxygen species (ROS) in both cellular signalling and damage [7]. The autophagy has also been implicated in the progression of diabetes, cancer, cardiovascular, neurodegeneration, immune diseases and ageing [8,9,10,11,12,13].

Mitochondria are the major source of ROS within cells [14,15] and mitochondrial ROS (mROS) are generally produced as by-products of the bioenergetics during oxidative phosphorylation (OXPHOS) [16]. The ROS are highly reactive metabolites of molecular oxygen (O_2_), including superoxide anion (O_2_^·−^) and hydrogen peroxide (H_2_O_2_), which are formed by electron reductions of O_2_ [17]. In the presence of transition metal ions, the more reactive hydroxyl radical (OH·) is produced [18]. ROS can act as signalling molecules at the physiological level, which contribute to various cellular processes, including proliferation, differentiation, programmed cell death, innate immunity, autophagy, redox signalling, calcium homeostasis, hypoxic stress responses and stem cell reprogramming [19,20,21,22,23,24,25]. Conversely, excess oxidative stress causes damages to proteins and cellular components, which is implicated in various pathologies [26].

Physiological ROS induce autophagy to maintain the cellular homeostasis in different types of cells, whereas dysregulation of redox signalling can demoralise the autophagic activity, which results in a variety of diseases [27,28]. However, the underlying mechanism between autophagy and redox signalling remains to be further elucidated. In this review, we introduce recent studies on redox signalling in autophagy regulation. Furthermore, we discuss the effect of autophagy on mitochondrial function and relevance to chronic pathologies.

## 2. Molecular Mechanisms of Autophagy

### 2.1. Autophagic Machinary

**Figure 1:**
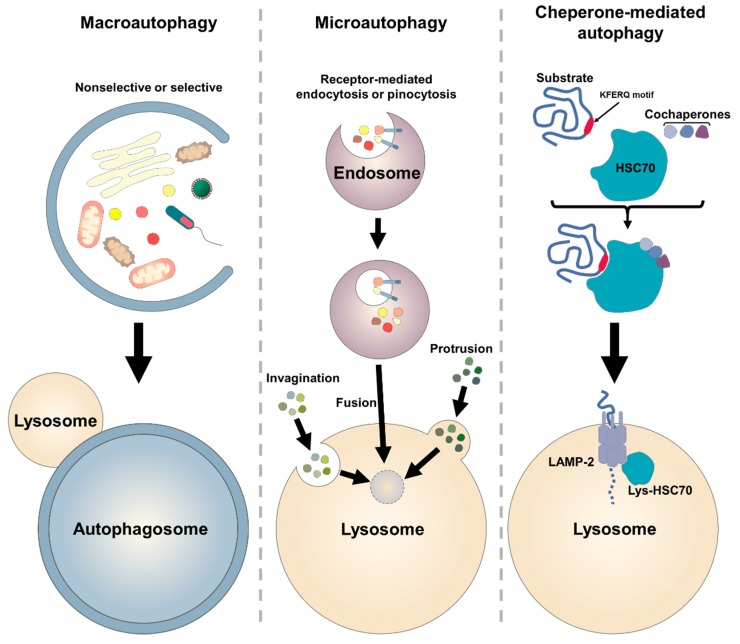
Overview of the mammalian autophagy pathway. Macro-autophagy encapsulates the cytoplasmic cargo by a delimiting membrane, which forms an autophagosome, which finally fuses with lysosome for degradation of the substrates. Micro-autophagy involves invagination or protrusion of the vacuole, which is formed by a lysosomal membrane. It also degrades extracellular molecules encapsulated by endocytosis (receptor-mediated pathway) or pinocytosis, following fusion with lysosome. The pinocytotic vesicles fuse with endosomes to hydrolyse the substrates. Chaperone-mediated autophagy is a selective degradation pathway, in which the protein substrates containing KFERQ-like motifs are recognised by chaperone HSC70 and cochaperones, such as carboxyl terminus of HSC70-interacting protein (CHIP), heat shock protein 40 (HSP40) and HSP70-HSP90 organizing protein (HOP), and are transferred into the lysosome via a lysosomal receptor complex, LAMP-2.

There are three major types of autophagy: (1) macro-autophagy, (2) micro-autophagy, and (3) chaperone-mediated autophagy (CMA) (Figure 1). Macro-autophagy is the most well-known form of autophagy. All types of autophagy promote degradation of damaged or functionally expired proteins and organelles in the cell.

(1) Macro-autophagy has been considered as a non-selective cellular process; however, this autophagy controls the quality of cellular contents via selective execution (e.g., long-lived proteins, aggregated proteins, damaged organelles, and intracellular pathogens) [29]. The autophagic pathway is initiated with the nucleation of a double-membraned structure, the phagophore (also known as isolation membranes), which is elongated to sequester the materials and to form a vesicle, autophagosome. The autophagosome is fused with lysosome to degrade the contents in the acidic environment. Then, the degraded molecules are recycled to the materials for rebuilding new cellular components [30].

(2) Micro-autophagy is a process that the cytoplasmic substances are directly engulfed into the lysosomes for degradation via entailing invagination, protrusion, or septation of the lysosomal or endosomal membrane [31,32]. Invagination of the endosomal membrane formed by endosomal sorting complexes required for transport (ESCRT) machinery incorporates the sequestered materials into the lysosome [33].

(3) Chaperone-mediated autophagy (CMA) is a form of autophagy present in various types of eukaryotic cell and tissue, but not in yeast [34]. A cytosolic chaperone, heat shock cognate protein of 70 kDa (HSC70), recognises that CMA-target proteins contain a pentapeptide motif biochemically related to KFERQ. The HSC70-target protein complex binds the lysosome-associated membrane protein-2A (LAMP-2A) on the lysosomal membrane, and then the target protein is translocated into the lysosomes to be degraded [35].

The present review focuses on the molecular and cellular mechanism, regulation, and selectivity of mammalian macro-autophagy (hereafter referred to as autophagy).

### 2.2. Molecular Biology of Autophagy

Autophagy induction is stimulated by diverse cellular events, including nutrient starvation, hypoxia, oxidative stress, pathogen infection and endoplasmic reticulum (ER) stress [36]. Autophagic multiprotein complexes are required for autophagic induction, which are hierarchically assembled and operated in the sites of the autophagosome formation termed pre-autophagosomal structure or phagophore assembly site (PAS) [37]. In mammalian cells, the autophagic process is initiated by inactivation of mechanistic/mammalian target of rapamycin (mTOR), which then requires the orchestration of several multiprotein complexes [38,39]. mTOR is a serine/threonine kinase that participates in a wide spectrum of biological processes [40]. It forms two functionally different complexes: mTOR complex 1 (mTORC) and mTORC2, which are structurally regulated by their modulators such as regulatory-associated protein of mTOR (Raptor), rapamycin-insensitive companion of mTOR (Rictor) and lethal with Sec13 protein 8 (LST8) via inter-complex and intra-complex loops [41]. However, mTORC2 is not responsible for controlling autophagy. Under normal condition, mTORC1 directly phosphorylates uncoordinated-51-like kinase 1 (ULK1), ULK2 and autophagy-related protein 13 (Atg13), of which both constitute an autophagy initiation complex via interaction with focal adhesion kinase family-interacting protein of 200 kDa (FIP200) and Atg101 [42,43]. ULK1 interacts with Atg13 and FIP200 at its C-terminal region [44] and binds to Atg101 through the N- terminus of Atg13 [45]. In response to starvation or stress conditions, the mTORC1 is dissociated from the ULKs complex via phosphorylations of Rheb and Raptor by AMP-activated protein kinase (AMPK) [46]. Subsequently Ulk1/2 are rapidly dephosphorylated and autophosphorylated, and Atg13 and FIP200 are phosphorylated [47]. The autophagic activation of ULKs complex contributes to phagophore nucleation [48].

The phagophore is a small cup-shape membrane structure, which elongates to form a complete autophagosomal structure, although its origin has still been debated [49,50]. In higher eukaryotic cells, it is accepted that at least under starvation conditions, phagophore nucleation occurs at the omegasome, morphologically resembling Greek capital letter omega (Ω), which is the region of endoplasmic reticulum (ER) enriched for phosphatidylinositol 3-phosphate (PI3) [51,52]. The formation of an omegasome requires Class III PI3 kinase (PI3KC3), which forms a complex with Beclin1, Beclin1-regulated autophagy protein 1 (AMBRA1), general vesicular transport factor (p115), p150 and ATG14L [42,53,54]. The ULKs complex leads to activation of the PI3KC3 complex via phosphorylations of Beclin1 and AMBRA1 [54,55,56]. The active PI3KC3 generates PIP3 via phosphorylation of PI on the surface of the phagophore, which recruits double FYVE domain-containing protein 1 (DFCP1) [57,58,59] and WD repeat domain phosphoinositide-interacting protein 2 (WIPI2) to mediate the nucleation and growth of the phagophore [60,61]. The activity PI3KC3 complex is also controlled via interacting with cofactors such as UV radiation resistance-associated gene (UVRAG), Bax-interacting factor 1 (Bif1) and RUN domain- and cysteine-rich domain-containing beclin-1-interacting protein (Rubicon) [30].

The phagophore is elongated to develop into an autophagosome, which is regulated by two ubiquitination-like conjugation systems: Atg5-Atg12 conjugation and microtubule-associated protein light chain 3 (LC3B) processing [62]. The Atg12 is activated by Atg7 (E1-like activating enzyme), and is then conjugated to Atg5 by Atg10 (E2-like conjugating enzyme) [63,64]. The Atg5-Atg12 complex interacts non-covalently with Atg16L1 (E3-like ligase enzyme), which results in the Atg5-Atg12-Atg16L1 multimeric complex [65]. Atg16L1 is recruited to the phagophore by physical binding with WIPI2 [66]. The Atg5-Atg12-Atg16L1 complex is associated with induction of curvature into the extending phagophore via asymmetric insertion of processed LC3B [67,68]. The Atg5-Atg12-Atg16L1 complex is recruited to the outer membrane of the phagophore, essentially avoiding premature fusion with the lysosome [69]. The C-terminal flanking region of nascent LC3B (proLC3B) is converted to LC3B-I via cleavage by Atg4 a cysteine protease. The exposed C-terminal glycine residue of LC3B-I is then activated by Atg7, and the LC3B-I is modified to LC3B-II through phosphatidylethanolamine (PE) conjugation by Atg3 [70,71]. The LC3B-II contributes to the autophagosomes closure [72], and the Atg5-Atg12-Atg16L1 complex is dissociated from completed autophagosomes [73]. The LC3B-II is anchored to the autophagosomal membrane until their fusion with lysosome. Then, the LC3B-II at the outer surface of the membrane is dissociated by Atg4 and recycled [74], whilst that remains associated with the membrane at the inner surface to degrade the substrates in the cargo [75]. An LC3-related protein, gamma-aminobutyric acid receptor-associated protein (GABARAP) has similar roles in the autophagosomal expansion process: autophagosome formation and substrate sequestration into double-membrane vesicles [76].

The phagophore extension is also supported by a transmembrane protein ATG9, which aids to supply lipid bilayers to the nascent phagophore, enabling further elongation of the autophagosome prior to closure of the fully formed autophagosome [50,77].

Tethering of the targeted cellular contents for degradation to an engulfing autophagosome is expedited by autophagy adaptor proteins such as sequestosome 1 (SQSTM1/p62) [78], optineurin [79,80], nuclear dot protein 52 kDa (NDP52) [81], neighbour of BRCA1 gene 1 (NBR1) [82] and autophagy-linked FYVE protein (ALFY) [83]. The completed autophagosome fuses to a lysosome to form autophagolysosome around centrosome via multiple proteins [84], including: syntaxin 17 (STX17) and synaptosomal-associated protein 29 (SNAP29) on the autophagosome; vesicle-associated membrane protein 8 (VAMP8) and vesicle transport through interaction with t-SNAREs 1B (VTI1B) on the lysosome [85,86,87,88,89]; and UVRAG [90]. The autophagolysosomal contents are finally degraded by lysosomal acidic hydrolases and released from the vesicle to cytoplasm, which produces building blocks and energy for cellular reorganisation and homeostasis [91,92].

## 3. Redox Signalling in Autophagy

### 3.1. Mitochondrial ROS and Redox Signalling

Reactive oxygen species (ROS) are small, short-lived and highly reactive molecules that are normally formed as by-products of oxygen metabolism in the mitochondrial electron transport chain (mETC) [93]. In the process of OXPHOS, electron leaks at complex I and III from mETC lead to the formation of partially reduced and highly reactive metabolites of molecular oxygen (O_2_), including O_2_^·−^ and H_2_O_2_, which are most important molecules in cellular signalling [94]. The mitochondrial O_2_^·−^ is catalysed to H_2_O_2_ by two dismutases including Cu/Zn-superoxide dismutase (Cu/ZnSOD) in mitochondrial intermembrane space (IMS) and cytosol, and manganese-dependent superoxide dismutase (MnSOD) in mitochondrial matrix [95]. The H_2_O_2_ can be converted to hydroxyl radical (OH·) by Fenton reaction [96]. Mitochondrial O_2_^·−^ also binds with hydrogen protons to form uncharged hydroperoxyl radical (HOO·) which react with unsaturated fatty acid of mitochondrial membrane lipids to generate lipid radicals [97]. Mitochondrial NO interacts with O_2_^·−^ to form RNS such as ONOO^−^, which causes cellular dysfunction by S-nitrosylating proteins [98]. Mammalian cells have numerous enzymes to degrade H_2_O_2_, including peroxiredoxins (Prxs), glutathione peroxidases (Gpxs), thioredoxins (Trxs) and catalase. Mitochondrial H_2_O_2_ is primarily eradicated by the action of Gpx1, Gpx2 and Gpx4, Prx3 and Prx5, Trx2 system, in which glutathione (GSH) is necessary [99,100,101]. Oxidized GSH (GSSG) is reduced to GSH by glutathione reductase (GR) [102]. Oxidized Trx2 is also recycled by Trx reductase (TrxR). The systems of H_2_O_2_ scavenging depend on nicotinamide adenine dinucleotide phosphate (NADPH) which is regenerated by three mitochondrial matrix enzymes: NADP^+^-linked isocitrate dehydrogenase (IDH), malate dehydrogenase (MDH) and nicotinamide nucleotide transhydrogenase (NNT) [94]. Catalase catalyses the breakdown of hydrogen peroxide to water and oxygen [103].

**Figure 2:**
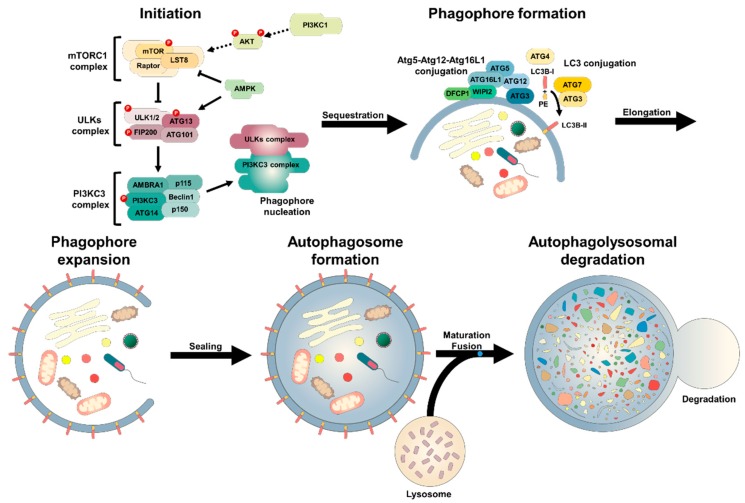
The general autophagy process. The autophagic process is divided into five distinct stages: Initiation, phagophore nucleation, autophagosomal formation (elongation), autophagosome-lysosome fusion (autophagolysosome) and cargo degradation. Autophagy is stimulated by various cellular stress conditions such as nutrient starvation and oxidative stress. Under stress conditions, mTORC1 is inhibited to activate the ULKs complex that encompasses ULK1/2, FIP200, ATG101 and Atg13. Subsequently, phagophore nucleation is induced by the activated ULKs complex, which is then mediated by the PI3KC3 complex that consists of multiple proteins, including Beclin1, AMBRA1, p115, p150 and ATG14L. The ULKs complex provokes activation of the PI3KC3 complex via phosphorylations of Beclin1 and AMBRA1. The active PI3KC3 produces PIP3 via phosphorylation of PI on the surface of the phagophore, which recruits DFCP1 and WIPI2 for phagophore nucleation and expansion. The phagophore is elongated to form the autophagosome, which is regulated by two ubiquitination-like conjugation systems: Atg5-Atg12 conjugation and LC3B-II conjugation. Atg12 is activated by Atg7, which is conjugated to Atg5 by Atg10. The Atg5-Atg12 complex interacts with Atg16L1. Atg16L1 is recruited to the phagophore via binding with WIPI2. The Atg5-Atg12-Atg16L1 complex is involved in the curvature of the elongating phagophore via asymmetric insertion of processed LC3B. The Atg5-Atg12-Atg16L1 complex is recruited to the outer membrane of the phagophore to avoid premature fusion of the autophagosome and lysosome. Nascent LC3B (proLC3B) is converted to LC3B-I via cleavage by Atg4, the exposed C-terminal glycine residue of LC3B-I is then activated by Atg7, and the LC3B-I is converted to LC3B-II via PE conjugation by Atg3. The LC3B-II is anchored to the autophagosomal membrane until the forming of autophagolysosome. Finally, the autophagolysosomal contents are degraded by lysosomal enzymes.

It has thus far been reported that the ROS are associated with autophagy induction upon deprivation of nutrients such as glucose, amino acids or serum [104,105,106,107]. Autophagy is activated in response to oxidative stress to protect the cells from apoptosis [108], whereas impairment of autophagy causes accumulation of oxidative stress [109]. Furthermore, antioxidant molecules moderately or completely suppress autophagic implementation [110]. Thus, mitochondrial ROS not only activate autophagic signalling but also inhibit it. In turn, the mROS and autophagy are mutually influenced. mROS generation and autophagic activation are summarized in Figure 2.

### 3.2. Autophagy Regulation by Redox Signalling

Mitochondria are generators and targets of ROS, which are inseparable from oxidative stress [109]. Accumulation of oxidative stress causes oxidisation and damage of cellular constituents, including proteins, DNA and lipids, which are oxidized and damaged which turn on the autophagic process [111]. Mitochondrial H_2_O_2_ has important roles in cellular signalling, which is rather stable than the other ROS molecules, and can easily diffuse to cytosol [112,113]. In response to nutrient starvation, the energetic stress possibly increases the demand of ATP production from mitochondria, which subsequently cause an increase of electron leaks, and thus relatively excess ROS are produced [114]. Indeed, mitochondrial H_2_O_2_ has long been implicated in the autophagic signalling pathway.

In response to nutrient starvation, H_2_O_2_ enables the reduced form of Atg4 to convert LC3B-I to LC3B-II through thiol modification of the Cys81 of Atg4, thus leading to increased autophagosome formation. However, the reduced form of Atg4 protease delipidates LC3 and inhibits autophagosomal membrane elongation, thereby suppressing autophagy [115]. Exogenous H_2_O_2_ also leads to oxidative stress and mitochondrial dysfunction, thereby inducing autophagy [116]. Treatment of H_2_O_2_ stimulated both autophagy and apoptosis in malignant glioma cells [117]. TNFα treatment increased ROS levels, and thus induced autophagy and cell death in Ewing sarcoma, which was also simulated by exogenous H_2_O_2_ treatment. These effects were reverted by chemical lipid radical scavengers or NF-κB activation [118,119]. Similarly, lipopolysaccharides (LPS) induce autophagy via H_2_O_2_ treatment [120]. O_2_^·−^ is also closely involved in autophagy induction under starvation conditions of glucose and amino acids [106]. Endogenous O_2_^·−^ levels were declined in a mETC-deficient (ρ°) cervical cancer cell line even under starvation conditions without endogenous levels of H_2_O_2_. Starvation-induced autophagy was considerably weakened in the ρ° cells [121]. Nutrient starvation also activated AMP activated protein kinase (AMPK), which inhibits mTORC1 activity and directly phosphorylates ULK1 at serine 317 (S317) and serine 777 (S777), thereby promoting autophagosome formation and autophagic flux [122,123]. AMPK also phosphorylated ATG13 at Ser224 to inhibition of autophagy, which elicits the intensity and duration of autophagy [124]. Starvation-induced AMPK activation was diminished in both ρ° or MnSOD overexpressed cells [121]. AMPK inhibitor compound C treatment or knockdown of AMPK catalytic subunit α1 also impeded starvation-induced autophagy [121]. AMPK-activated autophagy is modulated by ROS [125], in which upstream kinases of the AMPK are involved, leading to the induction of autophagy [126]. H_2_O_2_ directly activates AMPK by oxidizing cysteine residues of α and β subunits [127], or oxidation of ataxia–telangiectasia mutated (ATM) protein kinase [128]. Oxidative stress-activated ATM persuades its downstream signalling AMPK-Tuberous Sclerosis Complex 2 (TSC2) to suppress mTORC1, and thus induces autophagy [129,130]. Furthermore, in response to H_2_O_2_, AMPK is activated via phosphorylation at threonine 172 (T172) by liver kinase B1 (LKB1) kinase, which represses mTORC1, and thus induces autophagy [129].

NO is enzymatically synthesized from L-arginine by NO synthases (NOS) in oxidation processes [131]. In autophagic signalling, NO has different effects depending on the cell types. NO inhibits autophagosomal formation by attenuating the activity of S-nitrosylation substrates such as c-Jun N-terminal kinase 1 (JNK1) and inhibitor of nuclear factor κB (IκB) kinase subunit β (IKKβ). Starvation-derived autophagy is activated by JNK1 in mTOR-independent. JNK1 can phosphorylate B-cell lymphoma 2 (Bcl-2) to disrupt its interaction with Beclin1 for inducing autophagy [132,133]. IKKβ also induces autophagy by enhancing AMPK phosphorylation-dependent mTOR inhibition and JNK1-mediated Bcl-2 phosphorylation [134]. However, in glioma cells, inhibitory effects on the autophagic process were elicited by treatment of NO donors, such as sodium nitroprusside (SNP) and S-nitrosoglutathione (GSNO), following accumulation of LC3B-II [135].

It has increasingly been reported that the cross-talk between mROS and Ca^2+^ signalling has important roles in regulating autophagy. In response to hypoxia, mROS contribute to the transport of the stromal interaction molecule 1 (STIM1) to the plasma membrane, which activates Ca^2+^ release-activated Ca^2+^ (CRAC) channels, thereby inducing a Ca^2+^ influx increase and calcium/calmodulin-dependent protein kinase kinase 2 (CAMKK2) activation. Resultantly, AMPK and autophagy were activated [136,137]. Furthermore, mROS activate lysosomal Ca^2+^ channel mucolipin-1 (MCOLN1), which results in Ca^2+^ release and calcineurin-dependent nuclear translocation of transcription factor EB (TFEB), inducing Atgs and lysosomal proteins [138].

Nuclear factor erythroid 2-related factor 2 (NRF2) is a prominent transcription factor that regulates the gene expression of several genes encoding antioxidant and detoxifying enzymes, which maintain cellular redox homeostasis [139,140]. Kelch like ECH associated protein 1 (KEAP1) is a substrate adaptor protein within a larger E3 ubiquitin ligase complex containing cullin 3 (CUL3) and ring-box 1 (RBX1). It enables the ubiquitination and proteasomal degradation of the substrates, including NRF2 [141]. In response to oxidative stress, NRF2 is dissociated from KEAP1 and binds to an antioxidant-response element (ARE) sequence in the nucleus to activate its target genes [140]. In autophagic signalling, NRF2 induces p62 gene expression in response to oxidative stress, from which the protein further activates NRF2, forming a positive feedback loop [142,143,144]. Similarly, Sestrin2 leads to further activation of NRF2 [145]. Ubiquitinated p62 is phosphorylated, which enhances its affinity for KEAP1 to facilitate the autophagic degradation of KEAP1, thereby resulting in the stabilization of Nrf2 [146,147,148].

Tumour protein 53 (TP53 or p53)-induced glycolysis and apoptosis regulator (TIGAR) as a TP53 target interacts with hexokinase2 (HK2), which modulates the glycolysis pathway, thus upregulating NADPH production and reducing mROS levels [149,150]. Inhibition of TIGAR provokes mROS production and autophagy, whilst overexpression of TIGAR alleviates autophagy stimulated by nutrient deprivation or hypoxia in p53-independent [105]. Inhibition of TIGAR also induces mitophagy during ischemic injury, which is restored by antioxidant treatment [151]. Damage-regulated autophagy modulator (DRAM), a p53-regulated gene, also arouses autophagy [152]. Moreover, p53-induced Sestrin1 and Sestrin2 persuade autophagy via the activation of AMPK, thereby inhibiting mTORC1 [153].

### 3.3. Mitophagy

**Figure 3:**
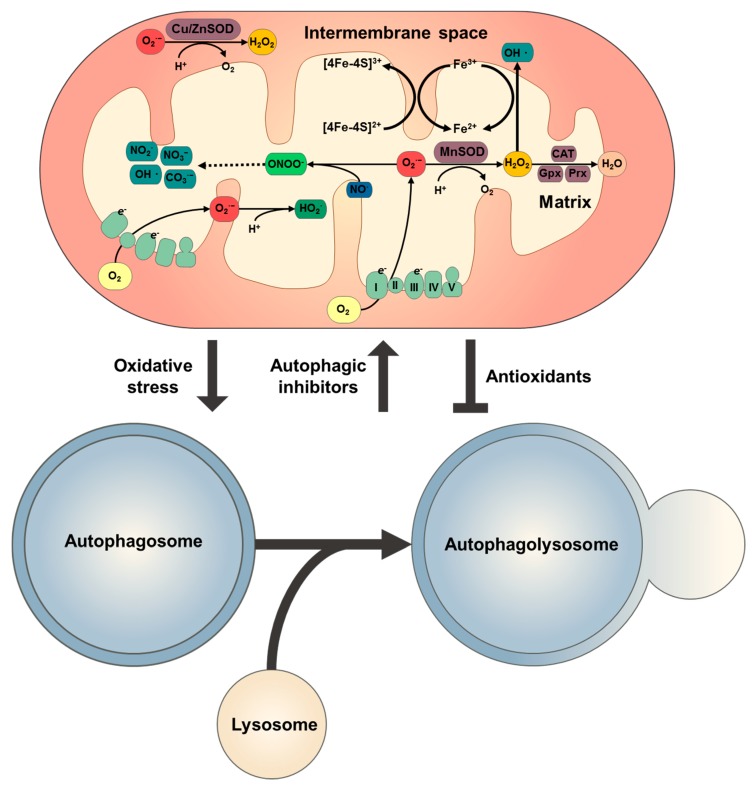
Interaction between mROS production and autophagy activation. Cu/ZnSOD and MnSOD catalyse the dismutation (or partitioning) of superoxide (O_2_^·−^) radical into hydrogen peroxide (H_2_O_2_) in the mitochondrial intermembrane space (IMS) and matrix, respectively. H_2_O_2_ is converted into water by catalase (CAT) and a group of glutathione peroxidases (Gpxs) and peroxirredoxins (Prxs). H_2_O_2_ react with redox-active ferrous ions (Fe^2+^) to generate the hydroxy radical (OH·) via the Fenton reaction. The H_2_O_2_ can easily be diffused to the other parts of the mitochondria or cytosol. The reaction between O_2_^·−^ and nitric oxide (NO·) produces peroxinitrite (ONOO^−^), which is decomposed to some highly oxidising intermediates including NO_2_^·^, OH·, and CO_3_^·^, and finally, to stable NO_3_^−^. O_2_^·−^ by itself can also reduce ferric iron (Fe^3+^) to Fe^2+^ in iron–sulphur centres of proteins, leading to enzyme inactivation and the concomitant loss of Fe^2+^ from the enzymes. Moreover, O_2_^·−^ can form the more reactive hydroperoxyl radical (HO_2_·) via protonation. The mROS-induced oxidative stress provokes autophagy, and autophagy inhibitors such as chloroquine (CQ) and bafilomycin A1 (BafA1) can further induce mROS production. Furthermore, antioxidants attenuate autophagic activation.

mROS are spontaneously gerarated during Mitochondrial ATP production by OXPHOS, which result in a certain degree of mitochondrial damage. The damaged mitochondria lead to ATP depletion and cytoplasmic cytochrome c (Cytc) release, which ultimately triggers activation of caspases and then apoptosis occurs [154,155]. To prevent cell death, the dysfunctional mitochondria are consequently removed from the mitochondrial network via selective autophagy, mitophagy [2]. Mitophagy can limit further production of mROS, which endorses mitochondria turnover and avoids dysfunctional mitochondria accumulation. Mitophagy is mainly regulated by the PTEN-induced kinase 1 (PINK1)-Parkin pathway, which is stimulated by MMP depolarization. PINK1 is a Ser/Thr kinase that translocates on the outer mitochondrial membrane (OMM), which is stabilized by low MMP, thereby causing mitochondrial depolarization [156,157,158]. PINK1 then recruits Parkin, which ubiquitylates OMM-located proteins, such as VDAC1, resulting in autophagic machinery recruitment and the selective sequestration of ubiquitylated mitochondria within autophagosomes [2]. Furthermore, the mitochondrial proteins, BCL2/adenovirus E1B 19-kDa interacting protein 3 (BNIP3) and NIX (Bcl-2/adenovirus E1B 19-kDa-interacting protein 3, long form, BNIP3L), contribute to mitophagy [159]. In response to oxidative stress after ischemia/reperfusion (I/R), BNIP3 is activated via homodimerisation, which induces mitophagy [160]. NIX, an atypical BH3 protein, is required for mitophagy in erythrocyte development. It directly recognizes autophagosome-sited GABARAP, and subsequently induces mitophagy [114,161]. ULK1 also regulates mitophagy via translocation to mitochondria to phosphorylate FUN14 domain containing 1 (FUNDC1) protein, an OMM protein, which is a receptor for hypoxia-mediated mitophagy [162]. Relationship between mROS and autophagy is schematized in Figure 3.

## 4. Pathological Implications

### 4.1. Cancer

Cancer cells exhibit continuous proliferation as a common trait, evading the suppression of growth and resisting cell death, during which metabolic activity increases via anaerobic metabolism, known as the Warburg effect [163]. This effect results in ROS production through incomplete OXPHOS. Moreover, cancer cells are exposed to the microenvironment of relatively low nutrients, oxygen (hypoxia) and pH, which lead to further ROS generation [164]. Thus, the mROS level is frequently increased in cancer cells, compared to normal cells [165]. In addition, treatment of chemotherapeutic agents or radiation induces mROS generation in cancer cells [166]. It is undoubted that autophagy is one of the defence mechanisms against oxidative stress. However, the autophagy regulated by mROS has distinctive beneficial and detrimental functions in cancer biology [167,168].

First, it is considered to have a tumour-suppressive effect during tumour initiation and malignancy progression, which contributes to removing damaged organelles and cells, thereby preventing cell proliferation and genomic instability [169]. Mutant p53 blocks the autophagic process by transcriptional inhibition of Sestrin1 and Sestrin2, which are activators of AMPK [170]. Likewise, mTOR as a nutrient sensor is involved in suppression of autophagy and promotion of proliferation in cancer cells, which is activated by glucose, amino acid, nucleotide, fatty acid, lipid, growth factors and hypoxia [171]. One of those, phosphatidic acid (PA), which is produced by catalysed hydrolysis of phosphatidylcholine via phospholipase D (PLD), can stimulate mTORC1 activation, thereby inhibiting AMPK in cancer cells [172,173]. Thus, controlling of PLD could be important to the efficacy of chemotherapeutic agents via facilitating autophagic pathways. Depletion of Beclin1 is frequently detected in various human cancers such as breast, prostate and ovarian cancers [174,175,176]. The loss of Beclin1 weakens autophagy induction, enhancing cancer cell proliferation. The attenuation of UVRAG or Bif1 also increases cancer cell proliferation via the impairment of autophagosome formation [177]. Epidermal growth factor receptor (EGFR) impedes autophagy by interacting with Beclin1, while the administration of the cetuximab drug inhibits EGFR via suppression of micro RNA 216b (miR-216b), which can prevent Beclin1 translation [178].

In contrast, autophagy plays roles in tumour progression, participating in the survival of cancer cells and the expression of oncogenes [179,180]. Although autophagy is inactivated during the initiation of tumorigenesis, it inclines to be restored tumour progression by allowing the cancer cells to achieve chemotherapeutic resistance [181,182]. Furthermore, autophagy enables cellular components to be recycled to supply metabolic substrates and removes damaged mitochondria in cancer cells [183,184]. Especially, transcription factor NRF2 is the major regulator of the antioxidant response in cancer cells [185]. The activation of NRF2 is associated to the poor prognosis of cancer patients by chemotherapeutic resistance via reducing the oxidative stress [185,186]. In cancer metabolism, NRF2 contributes to degradation of glutamine into glutamate, which provides cancer cells with nitrogen source to synthesize nucleotides and nonessential amino acids [186,187,188]. Furthermore, in response to oxidative stress, NRF2 induces autophagy via its noncanonical signalling pathway, transactivation of p62 gene, by which cancer cells avoid the apoptosis [142,143,189]. Activation of NRF2 undermines autophagy-targeted cancer therapy. Thus, a combination therapy to simultaneously targeting autophagy and NRF2 could be a good strategy in cancer treatment.

Cancer stem cells (CSCc) are a subtype of cancer cells, which have the abilities of self-renewal, and are directly associated with tumour initiation, chemoresistance, and metastasis [190]. Autophagy (mitophagy) is also closely involved in cancer stem cell survival via redox balance [191]. Autophagy is required for CD44^+^/CD24^low^ phenotype in breast CSCs, which is attenuated by LC3 or ATG12 knockdown or chloroquine treatment [192]. Autophagy has critical roles in transforming pancreatic cancer cells into CD133^+^ CSC-like cells under hypoxia [193]. Similarly, autophagy proteins, such as Beclin 1, ATG5 and ATG7, are increased in liver CSC CD133^+^ cells under hypoxic condition [194].

### 4.2. Diabetes

Diabetes mellitus, especially type 2 diabetes mellitus (T2DM), is one of the most common metabolic diseases, which is primarily involved in hyperglycaemia mitochondrial dysfunction, insulin resistance, lipid accumulation and abnormal regulation of autophagy [195,196,197,198]. ROS and oxidative stress are closely associated with the onset of diabetes and the development of complications [199].

Hyperglycaemia stimulates diacylglycerol (DAG)-protein kinase C-(PKC)-NADPH oxidase (NOXs) axis to accumulate ROS, which has been suggested to cause the progression of diabetes [200]. However, mitochondria have also been postulated as the main source of ROS in diabetes at the point that glucose is the main source of energy to operate the ETC during OXPHOS [14,201]. Furthermore, antioxidant enzymes are altered in two diabetic patients [202] with increased oxidative stress levels [203,204]. Autophagy (mitophagy) has cellular protective roles against the development of insulin resistance and increase in adiposity via alleviating mROS-induced oxidative stress [205].

Autophagy is repressed by chronic hyperglycaemia and subsequent insulin resistance. Pancreatic β cell line, Ins-1 cells exhibit apoptotic cell death via autophagy impairment by treatment of cathepsin inhibitors under high glucose conditions [206]. Autophagy is closely involved in the cellular architecture and function: Atg7 genetic ablation in pancreatic β cells causes islet degeneration and impairs insulin secretion, and Atg7 mutant mice exhibit glucose tolerance impairment and hypoinsulinaemia [207]. Moreover, autophagy is inhibited in streptozotocin-induced diabetic mice under high glucose conditions [208]. In diabetic hearts, autophagy is downregulated via inactivation of AMPK and subsequent JNK1-Bcl2, which fails to inhibit mTORC1 [209,210]. Downregulation of autophagic proteins has been observed in skeletal muscle from insulin resistance T2DM patients [211]. In adipose tissue, autophagy is also increased via attenuated mTORC1 activity [212]. In the liver, autophagy is inhibited in the presence of insulin resistance and hyperinsulinemia [213]. Although autophagy evidently has a beneficial role in insulin resistance and T2DM, the exact underlying mechanism of T2DM still remains to be elucidated.

Autophagy is also implicated in lipotoxic conditions. Cholesterol-induced ER stress exhibits increased autophagic flux in pancreatic β cells, facilitating LC3B-I conversion to LC3B-II. Cholesterol-induced autophagy was attenuated by treatment of chemical chaperone 4-phenylbutyrate (4-PBA) [214]. ER-stress induced autophagy can be regulated in mTORC1 independent [215]. In addition, glucolipotoxicity induces autophagy via TFEB in primary pancreatic β cells [216].

### 4.3. Neurodegeneration

Neurodegenerative diseases are closely associated with certain protein aggregates and abnormal autophagic process. Thus, autophagy plays important roles in the neurodegenerative pathology and therapeutics [217,218]. Autophagy is related to the maintenance and integrity of neuronal cells for the postmitotic nature of neurons [115,219]. It also reduces oxidative stress by removing unnecessary or damaged organelles and abnormal protein aggregates in injured neurons, which is helpful for cell survival [220]. Emerging roles of autophagy have been proposed involving antioxidant defence mechanisms for neuronal homeostasis [221]. Excessive oxidative stress-induced autophagy impairment has been proven to be implicated in the development of neurodegenerative diseases and their aggravation [222,223].

Alzheimer’s disease (AD) is one of the most common types of dementia; it is characterized by extracellular amyloid β (Aβ) plaques and intracellular tau (τ) protein tangles. Aβ is generated by the enzymatic cleavage of the amyloid precursor protein (APP) [224]. Oxidative stress is prominent in the pathogenesis of AD, related to the formation of Aβ plague, phosphorylation of τ protein and the formation of neurofibrillary tangles [225]. Autophagy participates in the degradation of Aβ [226]. The accumulation of Aβ results in an impaired fusion of autophagosomes with lysosomes [227]. Autophagy is involved in Aβ secretion into the extracellular space where it forms plaques. The deletion of ATG7 in APP transgenic mice results in downregulated Aβ secretion and plaque formation [228]. A mutation in Presenilin1 (PSEN1), which is involved in APP cleavage, illustrates one of the major characteristics of AD [229,230], and results in the impairment of lysosome function and the accumulation of Aβ [231]. PSEN1 also functions as an ER chaperone for the V0a1 subunit of lysosomal v-ATPase, of which the mutation impairs lysosomal v-ATPase maturation, and thus increases lysosomal pH [232]. Accumulation of τ protein into intracellular tangles is also a hallmark pathology of AD. Hyperphosphorylated τ protein co-localises with LC3B-II and p62 in patients with AD, as well as other neuronal disorders such as progressive supranuclear palsy (PSP) and corticobasal degeneration (CBD) [233]. Moreover, aberrant τ proteins disrupt axonal vesicle transport via the inhibition of complex, thereby increasing the number of autophagosomes in AD [234]. Furthermore, chronic or environmental stress, and increased glucocorticoid levels can induce AD and other pathologies [235,236]. Elevation of glucocorticoid levels could facilitate the formation of Aβ plague and phosphorylation of τ protein [235,237], which is mediated by mTOR-dependent inhibition of autophagy [238,239]. It is either associated with glucocorticoid-induced oxidative stress [240]. Recently, it has been reported that autophagy flux and stress granules could be monitored by RNA binding proteins (RBPs) such as T-cell intracellular antigen 1 (TIA-1), poly(A)-binding protein (PABP), Ras GTPase-activating protein-binding protein 1 (G3BP1), fused in sarcoma (FUS), and DEAD box 5 (DDX5) [241]. The level of these proteins is elevated in chronic stress and glucocorticoid responses. In addition, these RBPs are seemed to be related to oxidative stress responses and might be therapeutic targets for prevention of stress granule formation in AD and other τ pathologies.

As a movement disorder, Parkinson’s disease (PD) is characterized by the loss of dopaminergic neurons in the substantia nigra [242], which is pathologically related to oxidative stress, mitochondrial dysfunction and protein aggregation [243]. In PD, the autophagy pathway is impaired, leading to the accumulation of abnormal proteins [217]. Several genes are related to the initial pathology of PD, including PINK1, Parkin, α-synuclein and glucocerebrosidase (GBA) [244]. Autosomal recessive PD is associated with mutations in PINK1 and Parkin [245,246], which impair the degradation of damaged mitochondria via mitophagic activation [247]. Genetic ablation of Pink1 resulted in impairment of striatal mitochondria respiration and vulnerability to oxidative stress in the neuronal cells [248]. Similarly, deletion of parkin causes the dysfunctions of striatal mitochondria and synaptic plasticity [249,250]. PD is also characterised by intracytoplasmic bodies (Lewy bodies) that exist in the neuronal nucleus, which consist of an insoluble protein aggregate of α-synuclein that is degraded by CMA [251]. However, mutant α-synuclein has a high affinity with LAMP-2A, which impedes lysosomal uptake of the substrates, thus preventing CMA-dependent degradation [252]. Independent of the protein inclusions, an increased α-synuclein level impairs autophagy, which leads to the mislocalisation of ATG9 [253]. Moreover, GBA is one of the genetic risk factors of PD, of which homozygotic mutations cause the lysosomal disorder Gaucher disease [254]. A loss of GBA induces the accumulation of its substrate glucosylceramide within lysosomes, leading to autophagy impairment by lysosomal dysfunction [226].

Huntington’s disease (HD) is a neuronal disorder caused by mutant proteins with expanded glutamine repeats (polyQ) [255]. HD pathogenesis is strongly affected by neuronal autophagy dysfunction. Huntingtin (HTT) is the most studied polyQ protein, of which the mutation is observed in HD; it impairs cargo recognition by autophagosomes [256]. The wild type of HTT serves as a scaffold protein involved in recruiting several autophagy proteins to autophagosome in the selective autophagic process [257,258]. A loss of huntingtin decreases autophagosome transport and subsequently leads to the degradation of substrates [259]. Mutant HTT also inhibits a striatum specific protein, Rhes, which interacts with Beclin1 to process autophagy [260].

### 4.4. Cardiovascular Diseases

Autophagy at basal levels is necessary to maintain cellular homeostasis in cardiomyocytes [261]. The cardiomyocytes depend on the elimination of damaged proteins and dysfunctional organelles for their maintenance and survival [262,263]. In particular, cardiomyocytes are highly enriched in mitochondria; once damaged or exhausted, these organelles are rapidly removed by autophagy (mitophagy) degradation. Impairment of the degradation pathway induces high levels of mROS accumulation, leading to accumulation of protein aggregates, dysfunctional mitochondria and pathological cardiac remodelling [263,264]. Autophagy is associated with the development and progression of diverse cardiovascular diseases such as atrial fibrillation, cardiac hypertrophy, heart failure and ischemic/reperfusion (I/R) [197].

Danon disease (or glycogen storage disease Type IIb) is an X-linked lysosomal and glycogen storage disorder, which is associated with cardiac hypertrophy. In Danon disease, LAMP-2, which is required for autophagosome-lysosome fusion, is genetically deficient [265,266,267]. In transverse aortic constriction (TAC) models, myocardial deletion of Atg5 provokes cardiac hypertrophy, left ventricular dilatation and contractile dysfunction [268]. Moreover, Beclin 1 knockdown inhibits autophagosomal formation, and thus increased cell death in an I/R mouse model [269]. In chronic ischemia, autophagy and mitophagy are required for cardiomyocyte survival, evading tissue damages [270]. Vacuolar ATPase assembly of the integral membrane protein VMA21, a chaperone of V-ATPase, facilitates the proton pump and acidifies the organelles, of which mutation elevates lysosomal pH, thereby interrupting the autophagolysosomal degradation in X-linked myopathy with excessive autophagy [271]. The role of small RNAs has also been investigated in cardiovascular diseases related to autophagy. miR-199a induces cardiac hypertrophy by attenuating the autophagy via activation of mTOR, of which the overexpression results in the accumulation of p62 and inhibition of LC3B-II lipidation [272].

On the contrary, autophagy might play detrimental roles in the cardiovascular disease. Beclin1 haploinsufficient damps cardiac pathological remodelling, counteracting TAC-induced overload stress. Conversely, cardiomyocyte-specific Beclin1 overexpression magnified the pathological remodelling response [273]. Moreover, inhibition of Beclin1 by cardiac peptide urocortin attenuates cardiomyocyte cell death by excessive autophagic induction in I/R injury [274].

### 4.5. Immunity

Autophagy has important roles in immunity, which consequently influences inflammatory pathogenesis [12]. Autophagy eliminates invading pathogens via a selective pathway xenophagy in response to various types of infections [275]. The adapter proteins such as NDP52, optineurin and p62 are involved in xenophagy, which bind to the ubiquitinated proteins and further guide the autophagic proteins [276]. It is connected to various aspects of adaptive and innate immunities, including antigen presentation, cytokine and interferon production, and lymphocyte development [276].

Microbial infection activates the host immune system, in which autophagy can serve as a part of innate immunity, thereby eliminating the invading pathogens [12]. Inflammasomes are a cytosolic protein complex formed in response to the invading pathogens, which leads to processing and subsequent release of IL-1α, IL-1β, and IL-18. The inflammasome comprises an apoptosis-associated speck-like protein containing a caspase recruitment domain (ACS), the pro-Caspase1, and proteins for sensing microbial products including the nucleotide oligomerization domain (NOD)-like receptor (NLR) family of proteins, which includes NLRP1, NLRP3 and NLRC4 [277]. mROS and lysosomal damage can induce inflammasome activation, which is inhibited by clearance of the damaged organelles via autophagy [277].

The anti-microbial role of autophagy is also controlled by Type 1 helper T cell (Th1)/Th2 polarization. The Th1 cytokines provoke autophagy, while Th2 cytokines prevent it [278]. The pathogen-associated molecular patterns (PAMPS) on the surfaces of microbial pathogens activate autophagy via sensing by toll-like receptors (TLRs), by which the invading pathogens are destroyed [276].

Crohn’s disease is a type of inflammatory bowel disease (IBD), which is closely related to dysregulation of autophagy [279]. It is characterised by a single nucleotide polymorphism (SNP) in *ULK1*. Thus, the autophagic process is impaired during the disease. The mutations in the leucine-rich domain of nucleotide oligomerizing domain-containing protein2 (*NOD2*) are also associated with Crohn’s disease. NOD2 recruits ATG16L in the plasma membrane during bacterial invasion. The mutation in NOD2 persist the inflammation via impairment of autophagy induction and antigen presentation [280,281]. The SNP in Atg161L also attenuates autophagosomal formation in the disease [282,283]. An autophagy-related protein, microtubule-associated protein 1S (MAP1S), interacts with LC3B, and is involved in autophagosomal formation, which enhances the survival of intestinal epithelial cells via Wnt/β-catenin signalling in Crohn’s disease [284].

## 5. Concluding Remarks

In the current review, we have provided an overview of the general functions of mROS in autophagy and further introduced the pathological context. mROS are inevitably generated as the by-products of bioenergetics, which in turn forms part of the cellular nature. Further, they are directly or indirectly responsible as messengers for various cellular signalling pathways. Autophagy is a vital integrated biological process for cellular and organismic homeostasis. It allows spatial reorganisation and energy supply to cells via the orderly degradation machinery of unnecessary or dysfunctional components. Mounting evidence indicates that mROS represent the upstream modulators of autophagy. Thus, the interplay between mROS and autophagy is highly important to cellular homeostasis and survival. Autophagy basically has beneficial effects on mROS, which sense oxidative stress, and thus eliminate damaged or expired cellular constituents.

In pathology, a number of studies have also revealed the cross-talk between redox signalling and autophagy in the progression of diverse diseases. Excessive ROS production incurs the accumulation of oxidative stress, which is certainly implicated in chronic pathologies such as metabolic, neurodegenerative, cardiovascular, and immune diseases as well as cancers. Impairment or dysregulation of autophagic process causes mitochondrial dysfunction, thereby increasing mROS production. Of a certainty, autophagy has been inclined to decreases oxidative stress. In this aspect, autophagy restoration might be a therapeutic strategy for oxidative stress-related diseases. However, according to the cellular or tissue environments, autophagy rather exacerbates the diseases in response to mROS generation.

In summary, mROS-induced autophagy could be either a cellular protective mechanism that alleviates oxidative stress, or a destructive process. Therefore, it remains to further dissect the regulatory mechanisms of autophagy in redox signalling of diverse cellular physiologies and pathologies. Appropriate adjustment of autophagy would be crucial for the development of future therapeutic strategies for chronic pathologies responding to oxidative stress, based on the pharmacological modulation.
